# Impact of rehabilitation on mortality and readmissions after surgery for hip fracture

**DOI:** 10.1186/s12913-018-3523-x

**Published:** 2018-09-10

**Authors:** Dario Tedesco, Dino Gibertoni, Paola Rucci, Tina Hernandez-Boussard, Simona Rosa, Luca Bianciardi, Maurizia Rolli, Maria Pia Fantini

**Affiliations:** 1 Department of Biomedical and Neuromotor Sciences, University of Bologna, Via San Giacomo, 12, 40126 Bologna, Italy; 2 Department of Medicine, Stanford University, 1265 Welch Road, 94305, Stanford, California, USA; 3 Rizzoli Orthopedic Institute, Via Giulio Cesare Pupilli, 40138 Bologna, Italy

**Keywords:** Hip fracture, Elderly, Rehabilitation, Surgery

## Abstract

**Background:**

Hip fracture in elderly patients is a rising global public health concern because of population ageing, and increasing frailty. Long-term morbidity related to poor management of hip fracture is associated with decreased quality of life, survival, and increase in healthcare costs. Receiving postoperative rehabilitation is associated with better outcomes and a higher likelihood of returning to pre-existing level of functioning. However little is known about which postoperative rehabilitation pathways are more effective to optimize patient outcomes. Few studies have analyzed postoperative rehabilitation pathways in a universal healthcare system. The aim of this study is to analyze the impact of post-acute rehabilitation pathways on mortality and readmission in elderly patients undergoing surgery for hip fracture in a large metropolitan area in Italy.

**Methods:**

In this retrospective cohort study, we analyzed 6-month mortality from admission and 6-month readmission after hospital discharge in patients who underwent surgical repair for hip fracture in the hospitals of the Bologna metropolitan area between 1.1.2013 and 30.6.2014. Data were drawn from the regional hospital discharge records database. Kaplan-Meier estimates and multiple Cox regression were used to analyze mortality as a function of rehabilitation pathways. Multiple logistic regression determined predictors of readmission.

**Results:**

The study population includes 2208 patients, mostly women (*n* = 1677, 76%), with a median age of 83.8 years. Hospital rehabilitation was provided to 519 patients (23.5%), 907 (41.1%) received rehabilitation in private inpatient rehabilitation facilities (IRF) accredited by the National Health System, and 782 (35.4%) received no post-acute rehabilitation. Compared with patient receiving hospital rehabilitation, the other groups showed significantly higher mortality risks (no rehabilitation, Hazard Ratio (HR) = 2.19, 95%CI = 1.54–3.12, *p* < 0.001; IRF rehabilitation, HR = 1.66, 95%CI = 1.54–1.79, *p* < 0.001). The risk of readmission did not differ significantly among rehabilitation pathways.

**Conclusions:**

Intensive hospital rehabilitation was significantly associated with a lower risk of mortality compared to IRF rehabilitation and no rehabilitation. Our results may help in the development of evidence-based recommendations aimed to improve resource utilization and quality of care in hip fracture patients. Further research is warranted to investigate the impact of the rehabilitation pathway on other outcomes, such as patients’ functional status and quality of life.

## Background

Hip fracture in elderly patients is a global public health concern because of its frequency and functional consequences on individuals experiencing this event [1–3]. Population ageing and increasing frailty play an important role in delayed recovery and declining health in this group of patients [4]. Moreover, long-term morbidity related to poor management of hip fracture is associated with decreased quality of life and survival as well as significant increase in healthcare costs [5].

Evidence shows that delivery of surgery in hip fracture patients within 48 h of admission is associated with significant lower mortality rates [6–8]. International guidelines recommend an early mobilization of the patient on the day after surgery unless contraindicated and a post-acute rehabilitation plan led by a multidisciplinary team including physicians, physical and occupational therapists [9–11]. Receiving postoperative rehabilitation is associated with better outcomes and a higher likelihood of returning to pre-existing level of functioning [12–14]. However, little is known about which postoperative rehabilitation pathways are more effective to optimize patient outcomes and health system characteristics associated with better patient outcomes is scant [15–17]. In addition, to our knowledge few studies have analyzed postoperative rehabilitation pathways in a universal healthcare system [15].

Italy has a universal single-payer health system managed by the state and the regions. The central government oversees the overall system and defines the essential levels of care to be delivered to the citizens, free of charge or after the payment of a fee, with public resources collected through general taxation. The regions are responsible for the actual planning and delivery of services with a certain degree of autonomy [18, 19]. In the last decade, Emilia-Romagna region has improved the management of patients with hip fracture, issuing policies and guidelines aimed at reducing the delay of surgery and designing specific pathways for postoperative rehabilitation [20]. However, the effect of these programs at the population-level is unknown.

The aim of this study is to analyze the impact of post-acute rehabilitation pathways on mortality and readmission in elderly patients undergoing surgery for hip fracture in the large metropolitan area of Bologna, located in Emilia-Romagna region, Italy.

## Methods

### Study design and population

**Fig. 1:**
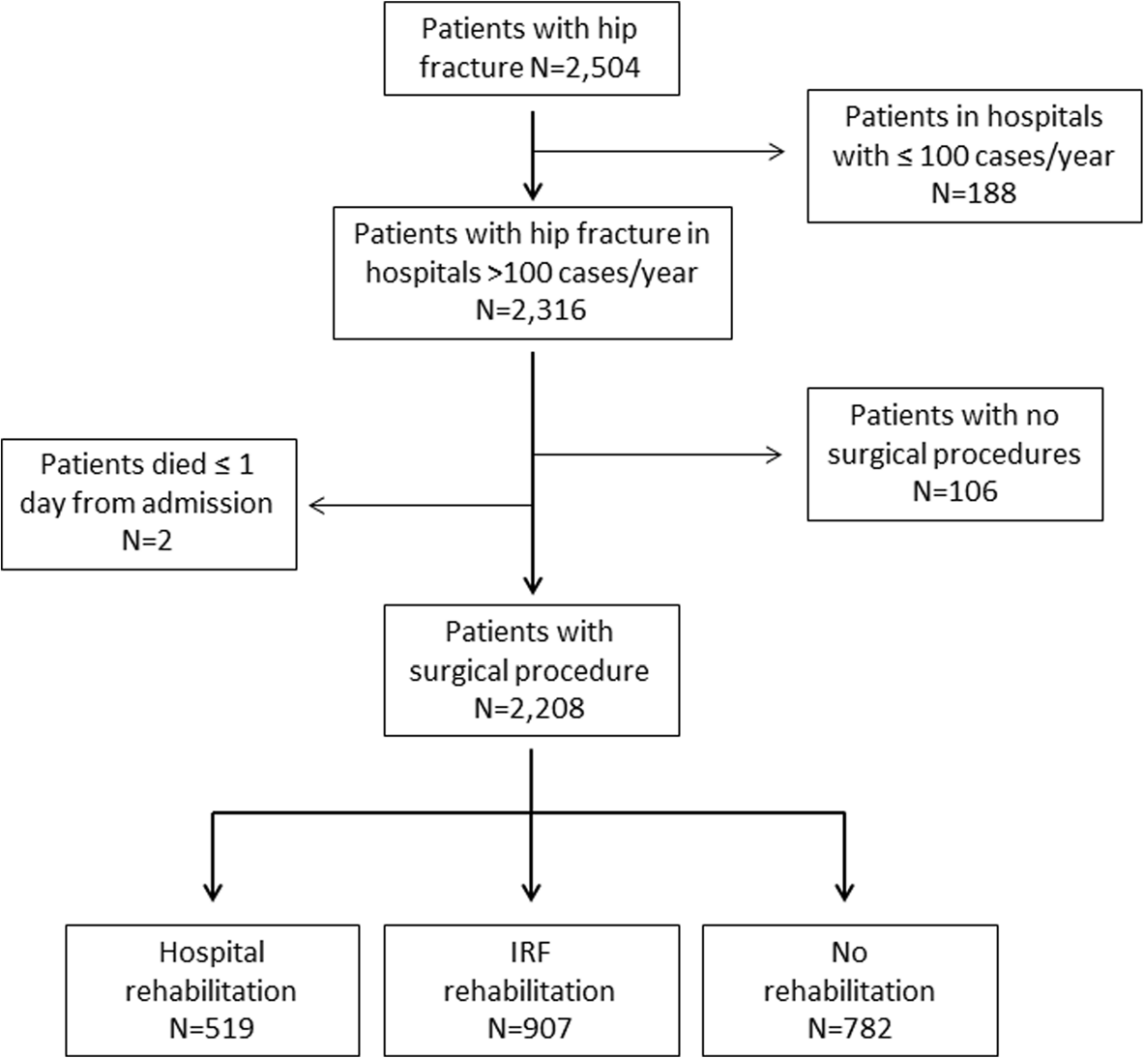
Flowchart showing patient selection in the study

**Fig. 2:**
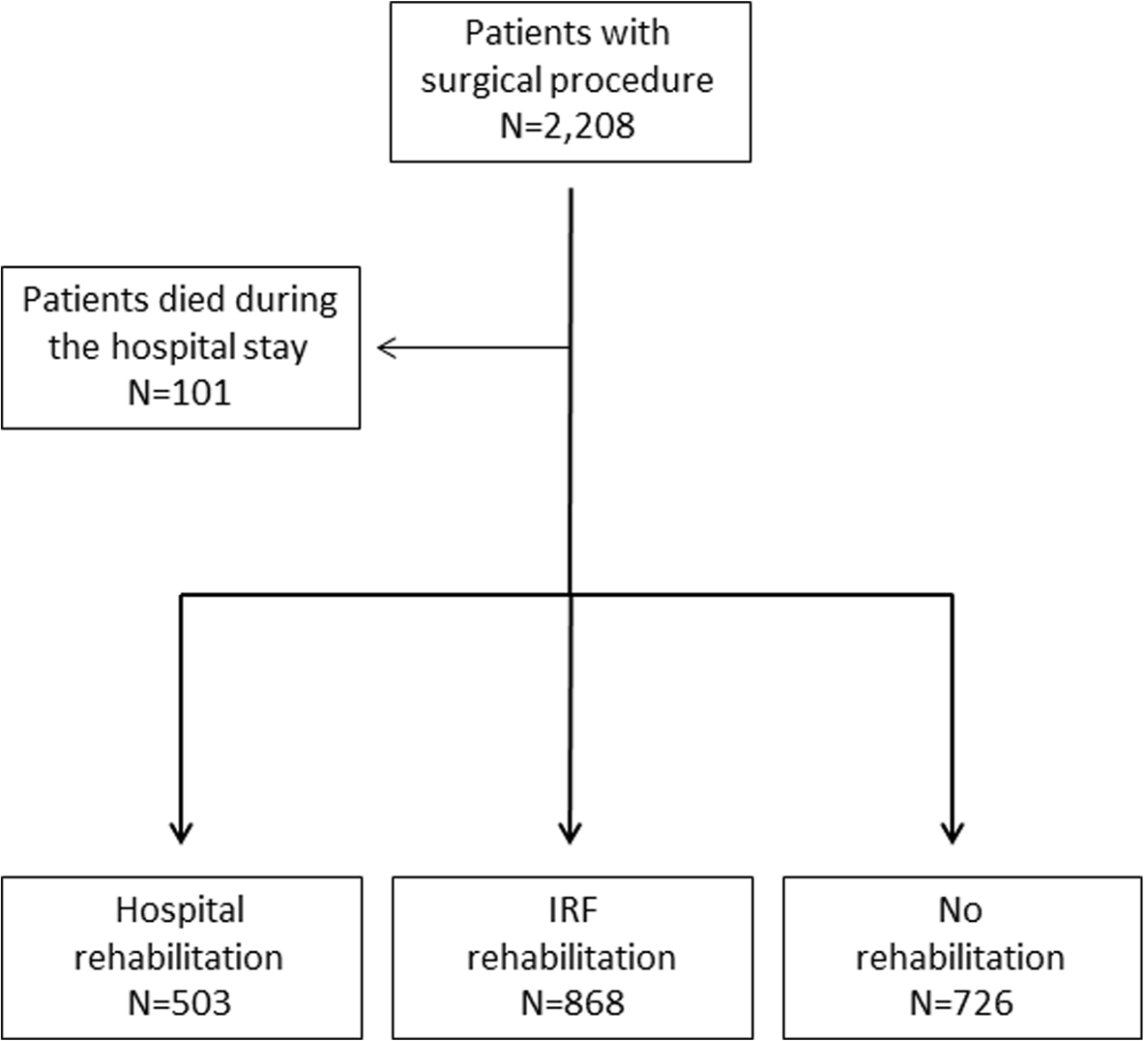
Flowchart showing patient selection for the analysis on readmissions

The data sources for this observational cohort study were the Hospital Discharge Records (HDRs) database of the Emilia-Romagna region (Italy), the mortality registry, the regional domiciliary care database (ADI) and outpatient specialty care database (ASA). We extracted from HDRs data for all patients aged > 64 years with a primary diagnosis of hip fracture (ICD-9-CM codes 820.0–820.9) from hospitals in the Bologna Metropolitan Area, Italy (over 1 million inhabitants and two Local Health Authorities), discharged between January 1, 2013 and June 30, 2014. We selected patients from hospitals with at least 100 procedures per year in order to reduce variations related to caseload, which resulted in the exclusion of 16 hospitals and 188 patients. We included patients who underwent a surgical procedure of total or partial hip replacement (ICD-9-CM codes 81.51–81.52) or the reduction of fracture and dislocation (ICD-9-CM codes 79.0–79.5), which resulted in the exclusion of 106 patients. We excluded patients who died within one day from admission (Fig. 1). For the analysis on readmissions we excluded patients who died during the hospital stay (Fig. 2).

### Interventions and outcomes

We considered the episode of care as the period of time between the admission for hip fracture and the discharge after post-acute inpatient rehabilitation, when provided. Patients were classified into three pathways according to the type of post-acute rehabilitation: 1) hospital rehabilitation, when rehabilitation was provided in the same hospital where patients received the surgical procedure; 2) IRF rehabilitation, when patients were admitted to private inpatient rehabilitation facilities (IRF), accredited by the National Health System, within 2 days from the index stay discharge and no previous post-acute rehabilitation had been performed. IRFs are private facilities that are accredited to provide inpatient post-acute rehabilitation within the National Health System and are reimbursed by the Region on the basis of a daily admission fee. The accreditation implies that IRFs are allowed to operate within the National Health System because they meet specific parameters of effectiveness and efficiency in terms of staff qualification, procedure volumes and quality of care provided.

We set a 2-day threshold for IRF rehabilitation in order to include patients who received inpatient care soon after the index admission. Over 90% of patients who received out-of-hospital rehabilitation were admitted to IRFs within 2 days from the index discharge; 3) no post-acute rehabilitation.

A small group of patients (~ 1%) who received both hospital and IRF rehabilitation were included in the hospital rehabilitation pathway.

Community rehabilitation was not included in our analysis because it is provided to only 9.6% of cases after discharge. Moreover, many patients seek private outpatient rehabilitation instead of community rehabilitation.

The outcomes of interest were mortality at 6 months from index admission, all-cause readmission, and orthopedic readmissions within 6 months from index discharge, in line with other studies [21–24].

Patients’ socio-demographic characteristics (age and gender) and comorbidity were also retrieved from hospital discharge records. Specifically, the presence or absence of myocardial infarction, heart failure, peripheral vascular disease, cerebrovascular disease, dementia, hypertension, neurological disease and renal disease was searched in the 2 years preceding the index admission using Elixhauser et al. algorithm [25]. The total number of comorbidities was also calculated. We used domiciliary care in the 6 months prior to the index stay as a proxy of patient frailty. Variables related to the admission included length of stay (LOS) from admission to discharge, including the hospital rehabilitation period, time to surgery (≤48 h, > 48 h), type of surgical procedure (reduction versus hip replacement), polytrauma (yes/no), open fracture (yes/no).

### Statistical analysis

χ^2^ and Kruskal-Wallis tests were used to compare patient characteristics among the three pathways. Following significant tests, post-hoc pairwise comparisons were performed at a Bonferroni-corrected significance level of 0.017 (0.05/3). Six-month mortality was estimated using Kaplan-Meier analysis and compared among rehabilitation pathways using log-rank test for the overall six-month period and then distinguishing the first 30-day period from the 31–180 day period. This was done to take into account the expectation that the majority of mortality events occur in the first month after admission. Multiple Cox regression was used to estimate the mortality risk in the rehabilitation pathways, adjusted for patients’ age, gender, comorbidities and intervention characteristics. The risk of all-cause and orthopedic readmission at 6 months in the three pathways, adjusted for the same set of covariates listed above, was estimated using multiple logistic regression models. In those models, the non-independence of patients within hospitals was taken into account by calculating robust standard errors adjusted for the patient-hospital clusterisation. Both in the Cox regression and in the logistic regression, a backward stepwise procedure was used to remove non-significant predictors until a parsimonious model including only significant covariates was achieved. Stata v.13.1 was used for all analyses.

## Results

Table 1 shows the characteristics of study patients, that included predominantly females and had a mean age of 84 years. Of the 2208 patients analyzed, 907, 41.1% received IRF rehabilitation, 519 (23.5%) hospital rehabilitation and 782 (35.4%) did not receive post-acute rehabilitation. The three groups did not differ significantly on age (*p* = 0.055) and gender (*p* = 0.542). Patients receiving hospital rehabilitation showed on average more comorbidities (3.4 vs. 2.1 for IRF rehabilitation and 2.2 for the no rehabilitation group, *p* < 0.001) and had a higher percentage of all-cause hospital readmissions at 6 months after discharge (31.8% vs. 23.3% for IRF and 23.7% for no rehabilitation, χ^2^ = 14.6, *p* = 0.001). In particular, patients receiving hospital rehabilitation had more frequently heart failure, peripheral vascular diseases, cerebrovascular disease, neurological diseases (other than dementia), and renal diseases (*p* < 0.001). Compared to the other groups, patients receiving IRF rehabilitation showed a lower incidence of cerebrovascular diseases and dementia (*p* < 0.001). Length of stay was significantly shorter in the no rehabilitation group (11.5 days) compared with the other two groups (35.2 and 34.2 days, *p* < 0.001). Patients receiving no rehabilitation showed a lower percentage of late (> 48 h) interventions (*p* = 0.017), more reduction procedures (*p* = 0.022) and a higher incidence of previous domiciliary care (*p* = 0.003). The percentage of patients with orthopedic readmissions was not different among subgroups (χ^2^ = 1.9, *p* = 0.376).

**Table 1:** Characteristics of the study population (*n* = 2208)

	Hospital rehabilitation (*N* = 519)	IRF rehabilitation (*N* = 907)	No rehabilitation (*N* = 782)	*p*-value	Significant post-hoc comparisons
Age, mean (SD)	83.8 (7.3)	83.7 (7.1)	84.5 (7.8)	0.055*	
N. comorbidities, mean (SD)	3.4 (2.0)	2.1 (2.1)	2.2 (2.0)	< 0.001*	hosp>IRF, no rehab
Length of stay, days (SD)	35.2 (18.2)	34.2 (13.6)	11.5 (7.2)	< 0.001*	hosp, IRF > no rehab
Sex, male, n (%)	134 (25.8)	211 (23.3)	186 (23.8)	0.542	
Myocardial infarction, n (%)	46 (7.1)	119 (5.1)	36 (4.6)	0.122	
Heart failure, n (%)	151 (29.1)	160 (17.6)	132 (16.9)	< 0.001	hosp>IRF, no rehab
Peripheral vascular dis., n (%)	185 (36.6)	216 (23.8)	148 (18.9)	< 0.001	hosp>IRF > no rehab
Cerebrovascular dis., n (%)	102 (19.7)	79 (8.7)	127 (16.2)	< 0.001	hosp, no rehab>IRF
Dementia, n (%)	112 (21.6)	113 (12.5)	170 (21.7)	< 0.001	hosp, no rehab>IRF
Hypertension, n (%)	28 (5.4)	74 (8.2)	50 (6.4)	0.111	
Neurological disease, n (%)	219 (42.2)	51 (5.6)	76 (9.7)	< 0.001	hosp>no rehab>IRF
Renal disease, n (%)	76 (14.6)	72 (7.9)	69 (8.8)	< 0.001	hosp>IRF, no rehab
Domiciliary care, n (%)	151 (29.1)	226 (24.9)	253 (32.3)	0.003	no rehab>IRF
Intervention > 2 days, n (%)	133 (25.6)	226 (24.9)	155 (19.8)	0.017	hosp, IRF > no rehab
Hip fracture reduction, n (%)	272 (52.4)	482 (52.1)	461 (58.9)	0.022	
Polytrauma, n (%)	34 (6.6)	59 (6.5)	53 (6.8)	0.973	
Open fracture, n (%)	6 (1.2)	70 (7.7)	45 (5.8)	< 0.001	No rehab, IRF > hosp
Died, n (%)	60 (11.6)	140 (15.4)	163 (20.8)	< 0.001	No rehab>hosp, IRF
Hospital readmission within 6 months, all causes, n(%)^a^	165 (32.8)	211 (24.3)	185 (25.1)	0.001	hosp>IRF, no rehab
Hospital readmission within 6 months, orthopedic, n(%)^a^	16 (3.2)	24 (2.8)	30 (4.1)	0.337	

Note: χ^2^ test was used, except where indicated. *Kruskal-Wallis test

^a^In the 2107 patients alive at the end of the episode of care

**Fig. 3:**
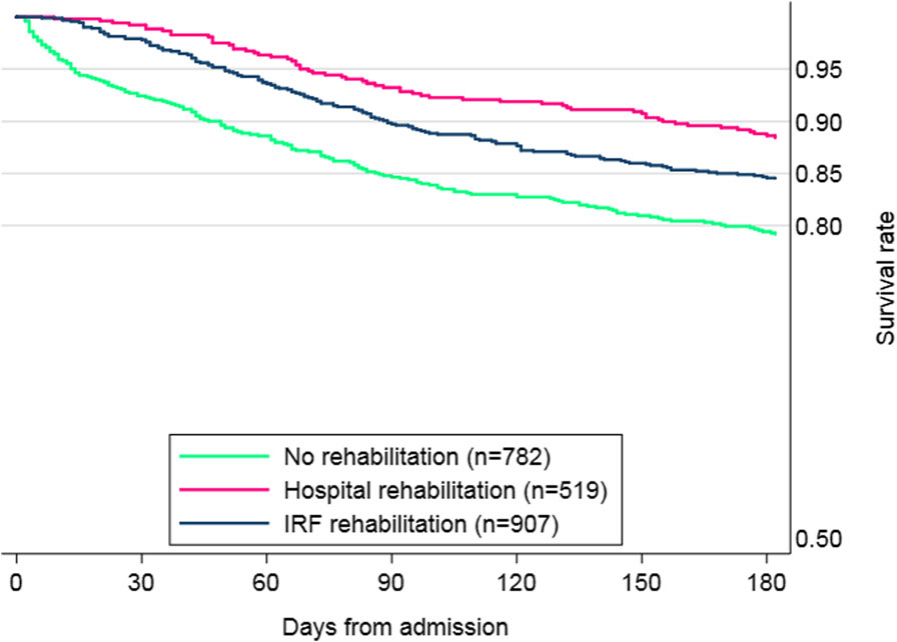
Kaplan-Meier survival at 6 months by rehabilitation group. Difference in 6-month survival among the groups was statistically significant (log-rank test: χ^2^ = 22.9, *p* < 0.001)

The cumulative 6-month mortality rate was 16.4%, with significant differences among the groups (χ^2^ = 20.7, *p* < 0.001). About one fifth (20.8%) of patients who did not receive rehabilitation died, compared with 15.4% of patients who received IRF rehabilitation and 11.6% of patients receiving inpatient rehabilitation. The Kaplan-Meier estimates of 6-month survival differed significantly among rehabilitation pathways (log-rank test = 22.9, *p* < 0.001), (Fig. 3). The survival gap among the groups occurred mainly in the first 30 days after admission, when mortality for the two rehabilitation pathways was 0.8% (hospital), 2.1% (IRF) and 7.5% in the no rehabilitation group (log-rank test = 7.0, *p* = 0.03). Between 30 and 180 days no significant difference in mortality was found among groups (log-rank = 3.5, *p* = 0.176).

Multiple Cox regression (Table 2) confirmed that the rehabilitation pathway predicted a differential mortality risk also after adjusting for covariates. Specifically, compared with hospital rehabilitation, the hazard ratio was more than twofold in the no rehabilitation group and 1.66 in the IRF rehabilitation group. The covariates independently predicting a significantly higher mortality at 6 months were male gender, older age, a higher number of comorbidities, myocardial infarction, heart failure, dementia, and intervention after 2 days from the hip fracture.

**Table 2:** Results of the Cox regression analysis of all-cause mortality risk at 6 months from admission on rehabilitation, patient, and inpatient stay characteristics

	HR (95%CI)	*p*-value
Hospital rehabilitation	1 [Reference]	[Reference]
No rehabilitation	2.19 (1.54–3.12)	< 0.001
IRF rehabilitation	1.66 (1.54–1.79)	< 0.001
Sex, male	2.01 (1.61–2.51)	< 0.001
Age, years	1.08 (1.05–1.11)	< 0.001
Number of comorbidities	1.06 (1.04–1.08)	< 0.001
Myocardial Infarction	1.37 (1.12–1.67)	0.002
Heart failure	1.26 (1.14–1.40)	< 0.001
Cerebrovascular disease	0.86 (0.74–0.99)	0.043
Dementia	1.22 (1.14–1.29)	< 0.001
Intervention > 2 days	1.27 (1.22–1.33)	< 0.001

Abbreviations. *HR* hazard ratio, *CI* confidence interval

The logistic regression of all-cause readmission at 6 months from discharge (Table 3) showed that the risk of readmission was similar across rehabilitation groups. Patients characteristics associated with a significantly higher risk of readmission were renal disease, length of stay, prior domiciliary care, dementia and number of comorbidities. Similarly, the risk of orthopedic readmissions at 6 months from discharge was unrelated to rehabilitation pathways (Table 4). Male gender and previous domiciliary care were associated with a lower risk of readmission, while length of stay, renal disease and neurological disorders other than dementia predicted a higher risk of readmission.

**Table 3:** Odds ratios of all-cause readmission within 6 months from discharge in the rehabilitation pathways, adjusting for patient and inpatient stay characteristics (*n* = 2107)

	OR (95%CI)	*p*-value
No rehabilitation	1 (Reference)	
Hospital rehabilitation	1.04 (0.83–1.31)	0.698
IRF rehabilitation	0.82 (0.56–1.22)	0.333
Sex, male	1.41 (1.20–1.65)	0.001
Age (years)	1.01 (0.99–1.03)	0.128
Number of comorbidities	1.11 (1.02–1.21)	0.012
Dementia	1.05 (1.01–1.09)	0.009
Length of stay	1.01 (1.00–1.01)	0.010
Renal disease	1.27 (1.00–1.62)	0.049
Domiciliary care	1.19 (1.09–1.29)	< 0.001

**Table 4:** Odds ratios of orthopedic readmission within 6 months from discharge in the rehabilitation pathways, adjusting for patient and inpatient stay characteristics (*n* = 2107)

	OR (95%CI)	*p*-value
No rehabilitation	1 [Reference]	[Reference]
Hospital rehabilitation	0.48 (0.19–1.24)	0.133
IRF rehabilitation	0.45 (0.20–1.04)	0.062
Sex, male	0.56 (0.47–0.66)	< 0.001
Age (years)	0.98 (0.95–1.01)	0.112
Length of stay (days)	1.01 (1.01–1.02)	< 0.001
Neurological disorders (other than dementia)	1.16 (1.01–1.34)	0.037
Renal disease	1.47 (1.01–2.14)	0.047
Domiciliary care	0.57 (0.46–0.70)	< 0.001

## Discussion

This is one of the first studies to assess the effects of different rehabilitation pathways following hip fracture surgery in a universal healthcare system. The results suggest that intensive post-acute rehabilitation within the index hospital was associated with lower risk of mortality compared to other pathways, including IRF rehabilitation, which was the most frequent pathway, although this does not imply a direct causation. Learning from these data, guidelines and recommendations could boost provision of post-acute rehabilitation in the best effective and cost-effective setting, possibly with standardized protocols.

Notably, the mortality risk in patients not receiving rehabilitation was more than twofold higher compared with patients who received hospital rehabilitation and it was 66% higher in those who received IRF rehabilitation, after adjustment for patient and intervention characteristics. These results are consistent with recent literature. Ireland et al. reported a 60% mortality risk reduction at 3 months and 40% at 1 year after hospital discharge in patients who received hospital-based rehabilitation compared with no rehabilitation [26]. Pitzul et al. reported similar results, with a majority of patients receiving postoperative rehabilitation in IRFs [15]. A possible explanation of the lower mortality in the hospital rehabilitation group of our study is that Italian hospitals have a multidisciplinary clinical approach to patients with hip fracture, which in some facilities includes orthogeriatric services. Orthogeriatric management of patients with hip fracture proved to be effective on frail populations and has already been adopted in the regional hospitals [14, 20, 27]. Zelzter et al., showed a 2.2% reduction of 30-day mortality in patients admitted to hospitals with orthogeriatric services compared to hospitals without orthogeriatric services [28]. Another study from Sweden showed that patients with hip fracture treated in hospitals with geriatric wards had lower risk of death and readmission [29]. In Italy, post-acute rehabilitation can be provided in third-level, high-specialized hospitals, as happens in 23.5% of patients of our cohort. Although our findings clearly support hospital-based rehabilitation, the healthcare system is not able to provide post-acute hospital rehabilitation to all patients. Other providers, such as IRFs, need to improve their quality of care, borrowing key aspects from the hospital organization, such as multidisciplinary teams, intensive rehabilitation and patient safety management plans.

Consistent with other studies, we found that mortality risk at 6 months was higher when surgery was performed more than 2 days after fracture, in males, and in patients with cardiovascular diseases or dementia, regardless of rehabilitation pathway [7, 30–34]. Our study found that a small proportion of patients receive surgery 2 or more days after the admission. In the last decade, Italy has successfully implemented national and local policies aimed at reducing the time to surgery for elderly patients with hip fracture [20, 35]. However, it is important to further enhance the access to timely surgery in this population, including patients 90 years of age or older, whose number is expected to significantly raise in the next future [35, 36]. Patients with dementia deserve particular attention, given the evidence showing that these patients can benefit from intensive postoperative rehabilitation programs [37, 38]. Seitz et al., showed that in patients with dementia, inpatient rehabilitation is associated with lower risks of long-term care admission and death compared with less intensive forms of rehabilitation, although in their population up to 40% of patients with dementia received no formal rehabilitation after hip fracture surgery [38]. Consistent with Seitz et al., our study showed that 43% of patients with dementia did not receive post-acute rehabilitation [38]. Another study by Mitchell et al., found that patients with hip fracture and dementia had higher mortality but could significantly benefit from intensive hospital rehabilitation [39]. Therefore, rehabilitation services need to be improved to ensure better quality of care for these patients. In our cohort, one third of patients did not receive rehabilitation and had significantly higher mortality than patients who underwent rehabilitation. Patients who did not receive rehabilitation showed a higher proportion of timely interventions (80%) compared with the other groups and one third of them received domiciliary care before the hip fracture, which can be considered a proxy of frailty. Although the Cox regression model adjusted patients’ survival for differences in comorbidities and demographic characteristics among groups, it is likely that other important clinical and psychosocial covariates not available account for the higher mortality of patients who were not rehabilitated.

Extending hospital rehabilitation services to all may not be feasible because of costs and lack of resources, [26] however, organizational efforts are needed to provide timely rehabilitation in adequate settings in order to increase the number of the patients receiving high-quality rehabilitation. Possible improvements could include providing the IRFs with shared orthogeriatic services and standardized rehabilitation protocols or implementing a large-scale program of intermediate care focused on rehabilitation services [27, 28]. Although intermediate care services may offer a great variety of inpatient services for an aging population, they should prove that these services are clinically effective and cost-effective [40, 41]. Further research is needed to understand which kind of post-acute rehabilitation care is more appropriate for specific subgroups of patients, for example patients with dementia or those over 85 years of age.

The different clinical pathways showed no significant differences in terms of all-cause and orthopedic readmissions. Male gender, length of stay and previous domiciliary care were the strongest predictors of all-cause readmissions. Orthopedic readmissions did not differ among subgroups. These findings may suggest that rehabilitation pathways have no impact on the long-term patients’ functional status.

Implementation of effective care pathways for patients with hip fracture is still debated, [24] and it is not clear which organizational characteristics are associated with better quality of care and outcomes. In order to provide the best possible match between healthcare settings and outcomes, further high-quality research on rehabilitation services in the hospitals and in other services is needed.

Our results should be interpreted in light of several limitations. First, that the study design does not allow to interpret associations between rehabilitation pathways and patient outcomes as causal relationships. Functional outcomes are not available in the administrative databases. A 12-month follow-up would have been more appropriate to study outcomes, such as mortality, still we had a specific focus on the episode of care, in which generally rehabilitation is completed, and there is a general agreement that the time span of the episode is 6 months. Third, administrative data can be affected by variability and lack of completeness in the coding of diagnoses and procedures. However, hospital discharge records from the Emilia-Romagna region showed good accuracy of ICD-9-CM coding, partially mitigating concerns about low coding standards [42–45]. Fourth, administrative data do not include functional status, which is a key outcome in the short and the long term. Fifth, the ‘no rehabilitation’ group may include patients who had access to private rehabilitation. More efforts to track these patients through the care pathways are needed. Finally, the small number of readmission events limited our ability to identify the predictors of readmission in logistic regression models.

## Conclusion

In conclusion, this population-based study of rehabilitation following hip surgery found that immediate rehabilitation within the index hospital was associated with lower risk of mortality. Still, we found that one-third of patients are discharged with no rehabilitation. Future programs focused on improved post-operative care should consider this evidence in planning and policy making. Innovative policies and health plans, aimed at targeting vulnerable populations, such as extremely older patients or patients with impaired cognitive functions, and implementing intermediate care models could be effective tools to expand the access to care and improve the quality of care in a growing high-need and high-cost population.

## Abbreviations

ADI: Domiciliary Care Database (*Italian:* Assistenza Domiciliare Integrata)
ASA: Outpatient Specialty Care (*Italian:* Assistenza Specialistica Ambulatoriale)
HDR: Hospital Discharge Record
IRF: Inpatient Rehabilitation Facility
LOS: Length of Stay
